# Tissue Oxygen Depth Explorer: an interactive database for microscopic oxygen imaging data

**DOI:** 10.3389/fninf.2023.1278787

**Published:** 2023-11-27

**Authors:** Layth N. Amra, Philipp Mächler, Natalie Fomin-Thunemann, Kıvılcım Kılıç, Payam Saisan, Anna Devor, Martin Thunemann

**Affiliations:** ^1^Department of Biomedical Engineering, Boston University, Boston, MA, United States; ^2^Department of Physics, University of California, San Diego, La Jolla, CA, United States; ^3^Department of Radiology, University of California, San Diego, La Jolla, CA, United States; ^4^Harvard Medical School, Martinos Center for Biomedical Imaging, MGH, Charlestown, MA, United States

**Keywords:** two-photon, phosphorescence, lifetime, metabolism, CMRO_2_

## Introduction

Over the last decade, increased efforts have been made to standardize the curation, storage, and retrieval of scholarly data (Data Citation Synthesis Group, 2014; Nosek et al., 2015; Wilkinson et al., 2016), including the heterogeneous data generated in experimental neurosciences (Rubel et al., 2022). Public availability of experimental data ensures independent validation of published studies beyond the peer-review process. Of equal importance, it facilitates the secondary use of experimental data, for a variety of applications such as generating or validating computational models without needing to reperform complex *in vivo* experiments. In this context, the FAIR guidelines ensure standards where data are Findable, Accessible, Interoperable, and Reusable (Wilkinson et al., 2016). Scientific journals and funding agencies promote, and in some cases mandate, data sharing within the scientific community. They achieve this by urging researchers to make their data accessible to the public and by establishing specific reporting criteria for frequently employed data formats (Kim et al., 2020; NIH, 2020; NSF, 2021; Science Magazine, 2023; SpringerNature, 2023) and by defining reporting requirements for commonly used data modalities. Nevertheless, in the case of less commonly employed experimental approaches, there is often a shortage of clearly established data standards. Consequently, researchers depend on individualized solutions to present their data in a manner that is accessible, comprehensible, and capable of being utilized by their peers. An example of this scenario can be found in the application of two-photon-based phosphorescence lifetime microscopy (2P-PLM) for quantitative oxygen measurements in live animals (Sakadzic et al., 2010; Lecoq et al., 2011)—a technique that is used by a small but growing community of researchers. Although based on standard two-photon laser scanning microscopy platforms, 2P-PLM generates more complex data and falls outside the scope of databases dedicated to standard microscopic imaging experiments.

Given these limitations, we developed “Tissue Oxygen Depth Explorer (TODE) v1.0,” a MATLAB-based database with graphical user interface (GUI) to simplify access and visualization of raw and pre-processed 2P-PLM oxygen imaging data. The datasets included with TODE v1.0 were generated as part of our recent 2P-PLM-based study on oxygen metabolism across cortical layers in awake mice under “resting-state” conditions (Mächler et al., 2022).

The cerebral metabolic rate of oxygen (CMRO_2_) is mostly dictated by metabolic demands of neuronal activity. It is a central parameter for the general understanding of brain energetics as well as the interpretation of blood oxygenation level-dependent magnetic resonance imaging (Heeger and Ress, 2002). From histological studies, it has been predicted that CMRO_2_ is higher in cortical layer IV than in layer I (Gonzalez-Lima and Cada, 1994; Weber et al., 2008). To address this prediction *in vivo*, we used 2P-PLM to measure tissue oxygen levels around penetrating arterioles at different cortical depths in awake mice under “resting-state” conditions (Mächler et al., 2022). Penetrating arterioles traverse the cerebral cortex from the surface toward the white matter and branch at variable depths to form a dense capillary network (Blinder et al., 2013). The exception is an area around penetrating arterioles largely devoid of capillaries resulting in prominent gradients from high pO_2_ close to the arteriole to low pO_2_ within the tissue. We had previously adapted the assumptions of the Krogh model to better represent this vessel geometry, allowing us to estimate CMRO_2_ at different cortical depths based on these pO_2_ gradients (Mächler et al., 2022). In contrast to previous predictions, we observed the highest CMRO_2_ in layer I and not in layer IV.

In the current paper, we present the MATLAB-based TODE database and GUI for easy access to the primary data generated in our recent study (Mächler et al., 2022). This application allows us to share these datasets (see structure of the database in Supplementary Tables 1, 2) in a form that facilitates close inspection by the research community and their use for alternative modeling approaches of cortical oxygen diffusion and metabolism.

## Methods

### Data collection and analysis

For animal preparation and further experimental details on data acquisition, including a schematic representation of the data collection process, please refer to the methods section and Figure 1A in Mächler et al. (2022). All experiments were approved by the animal care and use committees at UC San Diego and Boston University. Two-photon microscopy was performed on a commercial two-photon setup (Bruker) with femtosecond Ti:Sapphire laser (Coherent). The two-photon excitable phosphorescent dye Oxyphor 2P (Esipova et al., 2019) was provided by S. Vinogradov (University of Pennsylvania); a solution of Oxyphor 2P in artificial cerebral spinal fluid was pressure-microinjected through a silicone port (Roome and Kuhn, 2014) into the barrel cortex of awake mice. We added Sulforhodamine 101 labeling astrocytes to control for potential tissue damage due to the injection procedure. Vascular stacks were acquired at low (4× and 5× objective) and high (20× objective at 1× and 2× zoom) magnification after intravenous injection of commercially available fluorescein isothiocyanate (FITC)-labeled dextran or custom-conjugated Alexa 680-Dextran (Li et al., 2019). We performed 2P-PLM measurements at one or two penetrating arterioles in 1–2 sessions per animal at 0–500 μm below the cortical surface. Oxyphor 2P was excited at a wavelength of 950 nm at 400 individual points per depth plane that were oriented in radial or square grids around the penetrating arteriole. Oxyphor 2P phosphorescence was collected with a photon-counting Gallium Arsenide photomultiplier tube (Hamamatsu). During data collection, acquisition runs were divided into 20 iterations, in which 50 individual excitation-decay cycles of all 400 points were acquired each. Data was then transferred for post-processing into MATLAB (2019–2023). Some iterations were excluded if animal motion measured with an accelerometer exceeded a custom-set threshold during the acquisition of these iterations. The final phosphorescence decay profile was estimated from the sum of 500–1,000 acquisition cycles per point—depending on the number of excluded iterations. Non-linear least squares fitting (using the MATLAB function “lsqnonlin”) of a single exponential decay function was performed to estimate the decay time constant τ. Then, we used the Stern-Volmer equation to estimate pO_2_ (in mmHg) from τ using *in-vitro* calibration parameters measured for the respective Oxyphor 2P batches (Esipova et al., 2019). To normalize photon counts across measurements, per point, we divided the total photon count by the number of cycles to estimate photon counts/cycle.

### TODE database and GUI

The contents of the TODE database, i.e., individual datasets, were defined in a MATLAB file (“database.mat”) stored in the main data folder. This definition file contains a structural array with eleven rows – equivalent to the number of datasets stored in the TODE database—and 18 fields (described in Supplementary Table 1) containing metadata and dataset-specific settings for the GUI display. Individual datasets contain raw and processed phosphorescence decay data from Oxyphor 2P phosphorescence lifetime measurements at eleven penetrating arterioles in depths between 0 and 500 μm below the cortical surface. These datasets were generated from primary data stored by the imaging system using custom-written MATLAB scripts as described above and in Mächler et al. (2022). Note that phosphorescence decay profiles and photon counts stored here represent accumulated data after the exclusion of individual acquisition cycles potentially contaminated with motion (see above). In addition, the database contains image stacks of every penetrating arteriole visualized with intravascular FITC- or Alexa 680-Dextran, as well as low-magnification overviews of the surface vasculature. Image stacks were generated from primary imaging data stored by the imaging system; individual tiff files were combined into a multi-page tiff stack and converted from 16- to 8-bit grayscale to increase performance. Low-magnification overviews were created from maximum intensity projections of image stacks acquired at 4× or 5× magnification, Adobe Photoshop was used to highlight the artery of interest in the resulting image. The interactive database client GUI was created with the MATLAB App designer (MATLAB 2022a and 2023a; the source code and database releases are available here: https://github.com/NIL-NeuroScience/tode). The TODE GUI was packaged to run either as a MATLAB application or as a standalone application with MATLAB runtime libraries available for different platforms. An export option allows storing the data for each acquired cortical depth of each penetrating arteriole as a MATLAB data file for further use.

## Organization of TODE database and GUI

**Figure 1:**
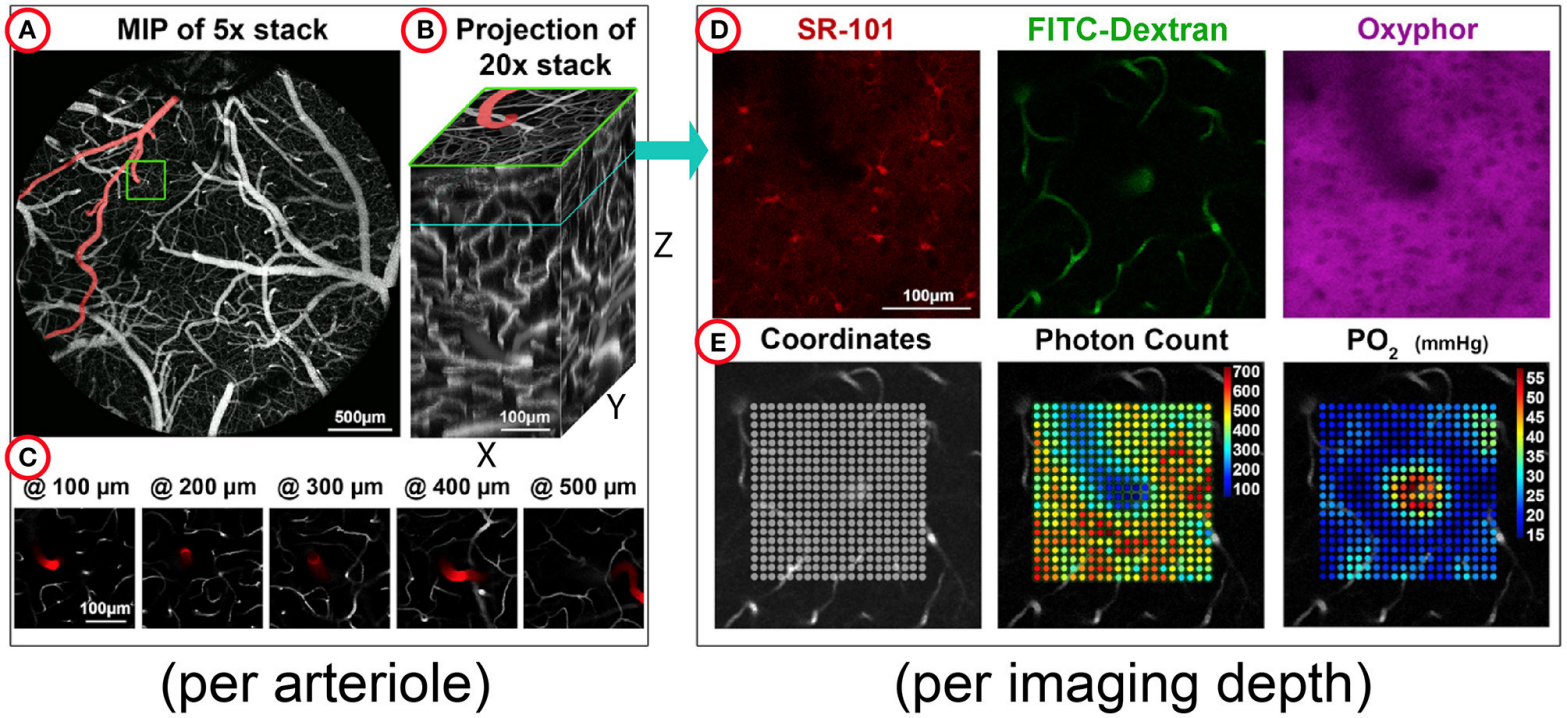
Overview of the structure of the microscopic oxygen imaging data stored in the TODE database. The experimental data in the TODE database quantitatively describe the oxygen landscape around individual diving arterioles in somatosensory cortex. The data were acquired at different depths below the surface in mice under “resting state” conditions. **(A)** The maximum intensity projection (MIP) of a Z stack at 5× magnification shows the top view of the exposed cranial cortex. The selected diving arteriole branching off a surface artery is highlighted by a green box. **(B)** Per diving arteriole, Z-stacks were acquired with a 20× objective and are shown here as *XZ, YZ*, and *XY* projections. The original Z stacks can be accessed through the TODE graphical user interface (GUI). **(C)** Individual Z planes show the depths where oxygen imaging was performed; the diving arteriole is artificially highlighted in red. **(D)** Distributions of Sulforhodamine 101 (SR-101), Fluorescein isothiocyanate (FITC)-Dextran, or Oxyphor 2P are shown for one selected depth. These images serve as reference images within the TODE GUI where they are superimposed with oxygen imaging data. **(E)** The FITC-Dextran reference image of the vasculature is superimposed with sampling coordinates (left), summative photon counts (center), or computed oxygen concentration values (“pO_2_”, right).

The organization of the experimental data is shown in Figure 1. Each penetrating arteriole in the database is stored in a separate directory containing two distinct subfolders: “pO_2_” and “stacks.” The “pO_2_” subfolder stores individual MATLAB files with acquisition parameters, reference images, and results from individual 2P-PLM acquisition, which runs at 5–6 discrete cortical depths. Reference images are two-photon images acquired at the same depths and show vascular labeling with FITC-Dextran, distribution of the extracellular dye Oxyphor 2P in the tissue, and, when available, astrocyte labeling with SR101 (see Figure 1D for examples). Each MATLAB file also contains spatial coordinates for every point within the acquisition grid, phosphorescence decay curves, corresponding non-linear fit parameters, and estimated pO_2_ levels for each grid point (see Figure 1E for examples). The estimated pO_2_ values of all acquired depths have a minimum of 23 ± 11 mmHg, a mean of 38 ± 11 mmHg and a maximum of 67 ± 16 mmHg (mean ± standard deviation of minimal, average, and maximum values, respectively). A subset of grids is acquired in both square and radial patterns, with the latter providing a higher sampling density close to the central arteriole. For a detailed explanation of all variables provided, see Supplementary Table 2. In contrast, the “stacks” subfolder contains high-resolution image stacks, captured after labeling of the vasculature with dextran-conjugated dyes. Unlike the reference images housed in the “pO_2_” subfolder, the “stacks” subfolder features image data from the respective penetrating arteriole, spanning from the cortical surface down to Layer IV (320–500 μm) and, in four instances, extending up to 900 μm, with a step size of 3–10 μm.

**Figure 2:**
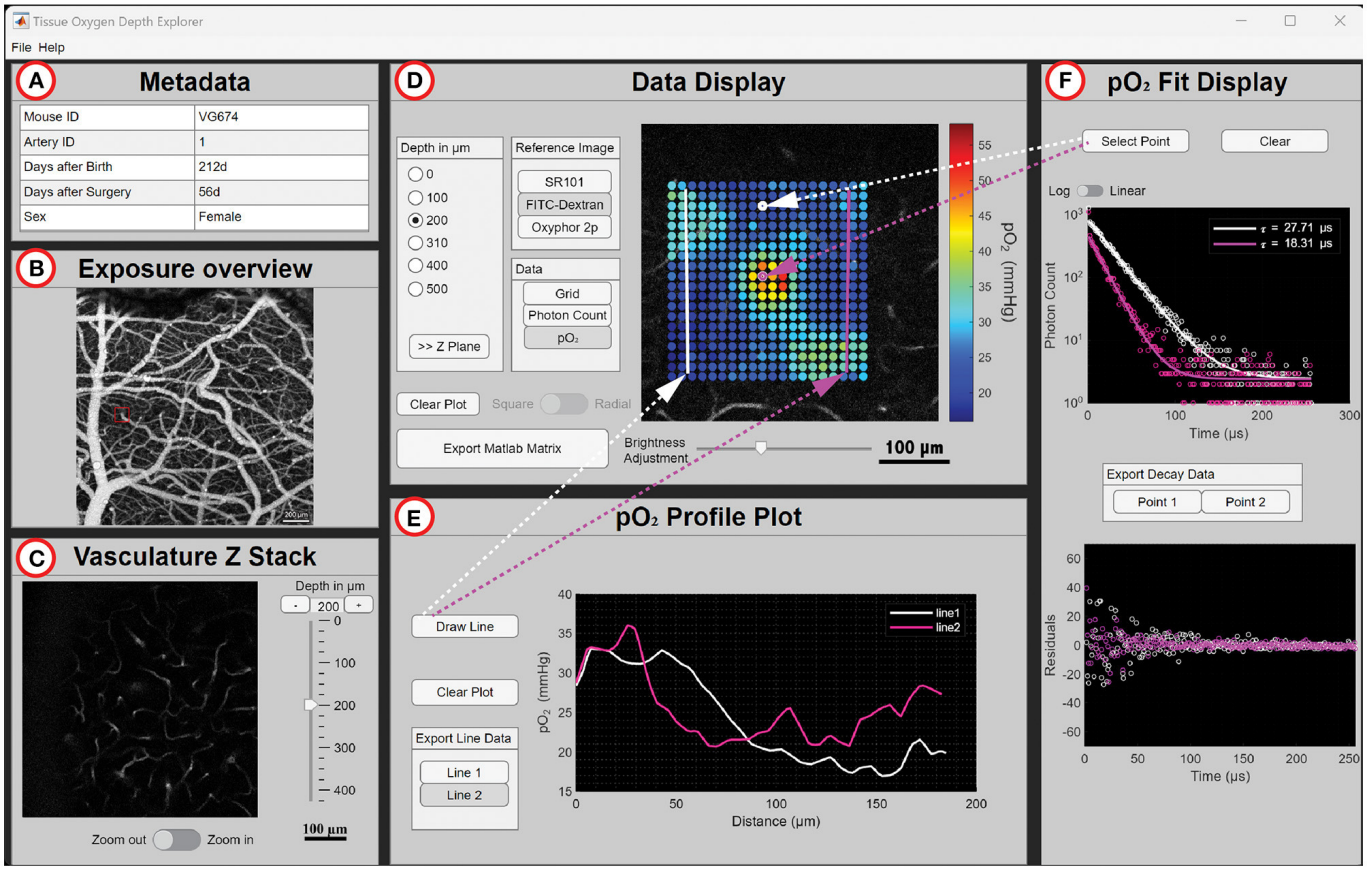
GUI organization and functionality are illustrated on an exemplary selection of the dataset. **(A)** Tabular overview of metadata on experimental animal and arteriole. **(B)** Overview maximum intensity projection of fluorescently labeled vessels. The red square indicates the target artery. The shadow on the top left indicates the port through which a pipette was lowered into the brain to deliver Oxyphor 2P and SR101. **(C)** Three-dimensional Z stack of the diving arteriole acquired at lower (Zoom out) and higher (Zoom in) magnification. **(D)** Data selection of a single arteriole measured at a depth of 290 μm. The white and pink lines represent the user-selected lines for which the interpolation profile plot is plotted in panel **(E)**. **(E)** The interpolated profiles of pO_2_ are plotted against distance along the line. **(F)** Top, phosphorescence decay for two points and the corresponding mono-exponential fits of the decays (lines) are displayed for two points the user can select in panel **(D)**. At the bottom, the residuals of the fits are shown.

We designed the TODE GUI to offer user-friendly access and visualization of the 2P-PLM oxygen imaging data. The layout of the GUI is shown in Figure 2; in the following, we provide a general introduction into the GUI and the functionalities of individual subpanels. The GUI includes a separate window that lists each dataset, each housing 2P-PLM measurements taken at various depths around one specific penetrating arteriole. Users can initialize the loading and display of a dataset from this panel. The application only loads the data corresponding to the selected artery and depth allowing for a seamless transition between datasets while limiting memory usage. In the main GUI, Figure 2A (Metadata) displays experimental detail, including animal ID, sex, age, and the interval between surgery and data collection. The metadata also provides the artery ID to distinguish datasets when several arteries from the same animal were included. Figure 2B (Exposure Overview) exhibits the vasculature labeled with fluorescent dextran as a maximum intensity projection of a two-photon image stack acquired with 4× or 5× objective. A red square highlights the penetrating arteriole from which 2P-PLM data was collected. Figure 2C (Vasculature Z Stack) presents two-photon image stacks of the penetrating arteriole labeled with fluorescent dextran from the cortical surface down to 400–900 μm acquired with a 20× objective, complete with a depth navigation slider and a zoom adjustment switch within the imaging plane. Figure 2D (Data Display) displays reference images along with phosphorescence lifetime imaging data. A slider allows brightness adjustment of the currently displayed reference image. The measurement depth can be chosen within the “Depth in μm” block. Here, the user can press the “>>Z plane” button to select the corresponding Z plane in Figure 2C. Once a depth is selected, the user can switch between different reference images in the “Reference Image” block. The “Grid” button in the “Data” block overlays the location of the acquisition grid on top of the reference image; the “Photon Count” button overlays a color-coded grid of photon counts at each data point; the “pO_2_” button overlays a colored-coded grid showing pO_2_ (in mmHg) at each data point. The switch button underneath the data block with labels “Square” and “Radial” allows the user to show data acquired in radial acquisition grids at selected locations if acquired at this location. The “Clear plot” button clears all plots overlaying the reference image. If some buttons are disabled for individual datasets, the respective data was not acquired. Figure 2E (pO_2_ Profile Plot) allows the user to draw one or multiple straight lines across the pO_2_ landscape shown in Figure 2E. An interpolated line graph is created with distance (*X*, in μm) vs. pO_2_ value (*Y*, in mmHg). After selecting the “Draw Line” button, the user draws a line in the reference image of Figure 2D. Several lines can be drawn by repeatedly choosing the “Draw Line” button. With the “Clear plot” button, the lines in Figure 2D, as well as the plots in Figure 2E, will be erased. In Figure 2F (pO_2_ Fit Display), an individual data point from the grid in Figure 2E can be selected after pressing “Select point,” and the top graph panel will show the photon count (*Y*) and the resulting curve from fitting a mono-exponential decay to the data vs. time (*X*, in μs). The bottom graph panel shows the residuals (*Y*) vs. time (*X*, in μs) for the mono-exponential fit. The “Clear plot” button clears the plots in Figure 2F.

The “Export MATLAB Matrix” button allows the users to save the 2P-PLM data for the chosen arteriole at the selected depth as a MATLAB file. The exported data consists of a structure array for the acquisition run corresponding to the artery at one depth level. The “Export Line Data” button, and the “Export Decay Data” allows the users to save the XY data of the graph. An example quality analysis of exported fitting parameters is given in Supplementary Figure 1 for the dataset shown in Figure 2.

## Benefits of TODE for oxygen imaging dataset sharing

Over the last decade, increased efforts have been made to standardize curation, storage, and retrieval of experimental data (The White House Office of Science Technology, 2013; Lee and Stvilia, 2017; NIH, 2020; Siminski et al., 2021). Public data availability ensures independent validation after publication and facilitates secondary use without the need to reperform experimental studies (Ascoli, 2015). However, researchers are forced to devise individualized solutions for sharing data from niche experimental modalities like 2P-PLM-based oxygen imaging that lack universally endorsed data standards. To address this, we introduced the TODE database and GUI in MATLAB, aimed at streamlining the access and visualization of 2P-PLM oxygen imaging data procured in our recent study (Mächler et al., 2022). This initiative aligns with our previous endeavors, where we facilitated access to 2P-based dynamic vascular imaging data through MATLAB-centric solutions (Sridhar et al., 2014; Uhlirova et al., 2017). TODE organizes individual acquisitions in a way that allows for efficient addition of data acquired in future studies or the adaptation of the application to similarly organized datasets.

TODE operates within MATLAB or as a platform-independent stand-alone application which does not require purchasing a MATLAB license. The TODE GUI balances the need for database tools to store and navigate through complex data as well as for tools for efficient data visualization. It enables the customization of data display and data export for other applications. MATLAB-based databases are less complex and more relatable to researchers in neuroscience and related fields compared to those using dedicated database languages such as Structured Query Language (SQL). Given the relative popularity of MATLAB in the field, data can directly be used for further analyses or modeling studies. These could involve computational reconstruction (“graphing”) of the microvascular network (Sakadzic et al., 2010; Gagnon et al., 2015), applying alternative model assumptions (Saetra et al., 2020), or comparison of our *in vivo* data on oxygen distribution with data from post-mortem structural analyses (Ji et al., 2021). Exemplarily, the latter study predicted a universal drop in pO_2_ between the capillary wall and the locations with the lowest pO_2_ of 15.9 ± 0.3 mmHg (mean ± SE) (Ji et al., 2021), a value that can be validated using the linear interpolation tool within TODE, as illustrated in Figure 2E, where ΔpO_2_ along the two lines drawn within the capillary bed was 16.1 and 15.3 mmHg, respectively. These modeling studies will contribute to physiologically founded bottom-up models of vascular and hemodynamic changes in response to changes in neuronal activity. They offer insights into cerebral blood flow regulation and non-invasive neuroimaging signals in humans (Gagnon et al., 2015; Uhlirova et al., 2016).

In summary, the TODE database and GUI exemplify tailor-made platforms for projects that involve ever-changing data processing streams common in cutting-edge experimental technologies.

## Data availability statement

The datasets presented in this study can be found in online repositories. The names of the repository/repositories and accession number(s) can be found at: https://github.com/NIL-NeuroScience/tode.

## Ethics statement

The animal study was approved by University of California San Diego IACUC and Boston University IACUC. The study was conducted in accordance with the local legislation and institutional requirements.

## Author contributions

LA: Conceptualization, Data curation, Software, Visualization, Writing—original draft. PM: Conceptualization, Methodology, Validation, Visualization, Writing—original draft, Writing—review & editing. NF-T: Data curation, Formal analysis, Validation, Writing—original draft. KK: Software, Validation, Visualization, Writing—original draft. PS: Methodology, Software, Writing—original draft. AD: Conceptualization, Funding acquisition, Supervision, Writing—original draft, Writing—review & editing. MT: Conceptualization, Software, Supervision, Validation, Visualization, Writing—original draft, Writing—review & editing, Data curation.
