# Supplementary material for: Tissue Oxygen Depth Explorer: an interactive database for microscopic oxygen imaging data
Source: Front Neuroinform. 2023 Nov 27;17:1278787. doi: 10.3389/fninf.2023.1278787 (PMC10711099; doi:10.3389/fninf.2023.1278787)
Supplement: Supplementary file 1 [file Data_Sheet_1.docx]

Supplementary Information for **“Tissue Oxygen Depth Explorer (TODE): An Interactive Database for Microscopic Oxygen Imaging Data”** by Layth N. Amra, Philipp Mächler, Natalie Fomin-Thunemann, Kıvılcım Kılıç, Payam Saisan, Anna Devor, Martin Thunemann_­_.

**Supplementary Table 1.** Overview of the variables defining the individual datasets stored in “database.mat” definition file. “Low-magnification” and “high-magnification Z stack” refer to Z stacks at 20× magnification and 1× and 2× zoom, respectively. “N” stands for the number of tick labels that vary dependending on the absolute depth of the Z stack.

| ***Variable*** | ***Size*** | ***Type*** | ***Unit*** | ***Description*** |
| --- | --- | --- | --- | --- |
| ID | 1×1 | string | [ ] | Dataset identifier |
| data_depth | 1×6 | double | [µm] | Depth below cortical surface where datasets were acquired. |
| data_hasRad | 1×6 | boolean | [ ] | Dataset with radial acquisition grid available |
| data_hasSquare | 1×6 | boolean | [ ] | Dataset with square acquisition grid available |
| hasFITC | 1×1 | boolean | [ ] | Dataset contains reference images with vascular staining with FITC-Dextran |
| hasOxyphor | 1×1 | boolean | [ ] | Dataset contains reference images with Oxyphor 2P staining |
| hasSR101 | 1×1 | boolean | [ ] | Dataset contains reference images with Sulforhodamine 101 staining |
| meta_Artery | 1×1 | double | [ ] | Artery ID (if more than one artery per animal was acquired) |
| meta_sex | 1×1 | char | [ ] | Sex of the animal |
| Z_depth | 2×1 | double | [µm] | Maximal depth in Z for low- and high-magnification Z stack |
| Z_inc | 2×1 | double | [µm] | Step size in Z for low- and high-magnification Z stack |
| Z_MajLabels | 2×N | cell | [ ] | Major tick labels of Z depth selection slider for low- and high-magnification Z stack |
| Z_MajTicks | 2×N | double | [µm] | Position of major ticks of Z depth selection slider for low- and high-magnification Z stack |
| Z_MinTicks | 2×N | double | [µm] | Position of minor ticks of Z depth selection slider for low- and high-magnification Z stack |
| Z_Offset | 2×1 | double | [µm] | Z offset (depth) for low- and high-magnification Z stack |
| Z_Overview | 1×1 | double | [ ] | Magnification of overview image |
| Z_slider | 2×2 | double | [µm] | Minimal and maximal depth in Z for low- and high-magnification Z stack |
| Z_Stack | 2×1 | double | [ ] | Number of planes across along Z for low- and high-magnification Z stack |

**Supplementary Table 2**. Overview of variables provided in the individual datasets of the TODE database. Variables are stored in one data file (.mat) per arteriole and depth. Two individual files are stored when data from the rectangular and circular acquisition grids are available. Abbreviations: n_Points_, number of points where phosphorescence decays were acquired (here: 400); n_Bins_, number of bins per decay (here: 2^8^ = 256).

| ***Variable name*** | ***Size*** | ***Type*** | ***Unit*** | ***Description*** |
| --- | --- | --- | --- | --- |
| **Main array: pO2** | | | | |
| calibration 1x1 struct [ ] Contains calibration parameters for the Stern-Volmer equation (y0, A1, t1, A2, t2) | 1×1 | struct | [ ] | Calibration of the Stern-Volmer equation (y_0_, A_1_, t_1_, A_2_, t_2_); t_1_, t_2_ in [s] |
| data | n_Bins_×n_Points_ | double | [ ] | Photon counts per time bin and point (sum across repetitions and iterations) |
| dbEntry | 1×1 | struct |  | Information on experiment and animal (*see below*) |
| fitC2 | n_Points_×1 | double |  | Parameter C_2_ (i.e., τ from mono-exponential fit) |
| fitDiag | n_Points_×1 | struct |  | Diagnostic information on mono-exponential fit (*see below*) |
| fittedCurve | n_Points_×1×n_Bins_ | double | [ ] | Fitted mono-exponential decay curve |
| pO2Value | n_Points_×1 | double | [mmHg] | pO_2_ value estimated from τ using Stern-Volmer equation |
| photonCountCols | n_Points_×3 | double |  | RGB tuple describing color of individual point in photon count grid |
| pixelSizeX | 1×1 | double | [µm] | Pixel size (resolution) of acquired data in X dimension |
| pixelSizeY | 1×1 | double | [µm] | Pixel size (resolution) of acquired data in Y dimension |
| PLIMCyclePeriod | 1×1 | double | [µs] | Period of single acquisition cycle |
| PLIMCyclesPerPoint | 1×1 | double | [ ] | Number of acquisition cycles per point |
| PLIMExcitationTime | 1×1 | double | [µs] | Duration of excitation gate |
| PLIMRepetitions | 1×1 | double | [ ] | Number of repetitions per acquisition cycle per point |
| PLIMTimeBin | 1×1 | double | [µs] | Temporal resolution of decay data |
| pO2Cols | n_Points_×3 | double |  | RGB tuple describing color of individual point in pO2 grid |
| PointsX | 1×n_Points_ | double | [ ] | Relative location of Points in × dimension |
| PointsY | 1×n_Points_ | double | [ ] | Relative location of Points in Y dimension |
| references | 1×3 | struct |  | Reference images (*see below*) |
| sum | n_Points_×1 | double | [ ] | Temporal sum of counted photons per point |
| tNew | n_Bins_×1 | double | [µs] | Time vector for photon decay data |
| **Sub-array: dbEntry** | | | | |
| date | 1×1 | string |  | Date of experiment |
| mouseID | variable | char |  | Animal identification (lab-internal) |
| mouseStrain | variable | char |  | Animal strain (lab-internal) |
| mouseDOB | 1×1 | string |  | Animal date of birth |
| mouseSurgery | 1×1 | string |  | Date of surgery |
| artery | 1×1 | double |  | Artery identification |
| **Sub-array: references** | | | | |
| info | variable | char |  | Dye (SR101, FITC, or Oxyphor 2P) |
| image | 512×512 | uint8 |  | Image data |
| **Sub-array: fitDiag** | | | | |
| cAll | 1×3 | double |  | Solution of the non-linear fit (C_1_, C_2_, C_3_) |
| resnorm | 1×1 | double |  | Squared norm of the residual |
| resid | n_Points_×1 | double |  | Residuals |
| output | 1×1 | struct |  | Information about the optimization process (refer to lsqlnonlin for reference) |
| lambda | 2×1 | struct |  | Lagrange multipliers at the solution |
| jacobinian | n_Points_×3 | double |  | Jacobian at the solution |


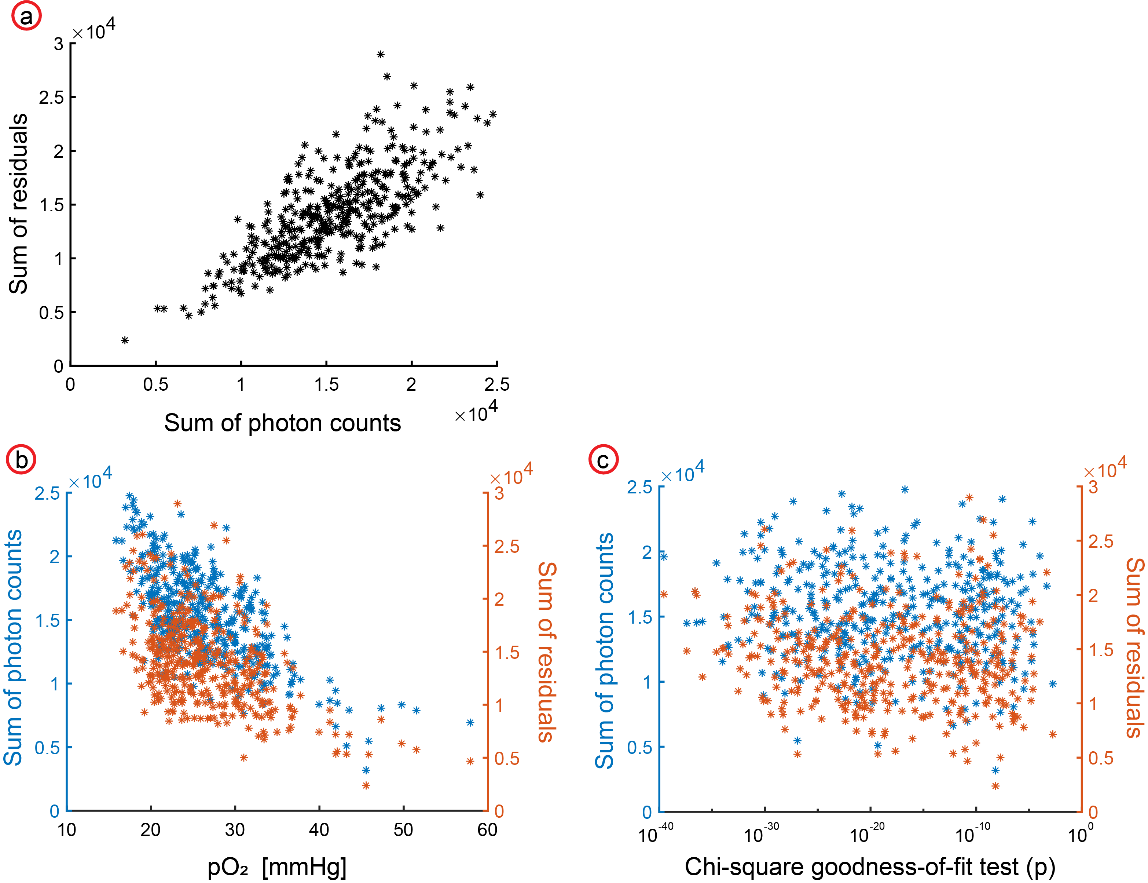


**Supplementary Figure 1**. **Further statistical analysis of the fitting procedure of phosphorescence data.** Data from 400 individual points of a square grid are shown; data was taken from the same measurement shown in Figure 2. a) The sum of photon counts used to estimate oxygen concentrations through non-linear fitting is plotted against the sum of normalized residuals. b) The sum of photon counts, and the sum of normalized residuals are both anticorrelated to estimated pO_2_ values. This can also be explained by a lower Oxyphor 2P signal close to light-absorbing vessels that also provide oxygen to the surrounding tissue. c) The sum of photon counts, and the sum of normalized residuals are not correlated with the p values of a chi-squared goodness-of-fit test, indicating that there is no impairment of the fit procedure due to lower photon counts and residuals within the measurement landscape.
